# Comparison of Survival Between Irreversible Electroporation Followed by Chemotherapy and Chemotherapy Alone for Locally Advanced Pancreatic Cancer

**DOI:** 10.3389/fonc.2020.00006

**Published:** 2020-01-22

**Authors:** Chaobin He, Xin Huang, Yu Zhang, Zhiyuan Cai, Xiaojun Lin, Shengping Li

**Affiliations:** ^1^State Key Laboratory of Oncology in South China, Department of Pancreatobiliary Surgery, Collaborative Innovation Center for Cancer Medicine, Sun Yat-sen University Cancer Center, Guangzhou, China; ^2^State Key Laboratory of Ophthalmology, Zhongshan Ophthalmic Center, Sun Yat-sen University, Guangzhou, China

**Keywords:** locally advanced pancreatic cancer, irreversible electroporation, chemotherapy, efficacy, SEER

## Abstract

Locally advanced pancreatic cancer (LAPC) has a dismal prognosis even after standard chemotherapy, and local progression contributes to nearly one-third of the deaths of these patients. As a local destructive method, irreversible electroporation (IRE) can feasibly treat LAPC. The aim of this study was to evaluate IRE combined with chemotherapy as a new treatment and compare its efficacy with that of chemotherapy alone in patients with LAPC. The data of LAPC patients who received chemotherapy with or without IRE were extracted from Surveillance, Epidemiology, and End Results (SEER) database and medical records of Sun Yat-sen University Cancer Center (SYSUCC). The efficacy of these two treatments was compared using propensity score matching (PSM) analysis. LAPC patients treated with the combination therapy had better overall survival (OS). Significantly higher cancer-specific survival (CSS) and progression-free survival (PFS) rates were also observed in patients after IRE combined with chemotherapy, compared with chemotherapy alone. IRE combined with chemotherapy was established as a favorable factor for OS, CSS, and PFS in LAPC patients. This combination method may be a more suitable treatment for patients with LAPC.

## Introduction

Pancreatic adenocarcinoma (PDAC) is the seventh leading cause of death in cancer patients (1), and locally advanced pancreatic cancer (LAPC) accounts for ~40% of all PDAC cases. Despite currently available therapies, the 5-year overall survival (OS) rate of LAPC patients was only 3% and the median OS were only 11 months (2). Palliative chemotherapy remains the recommended therapy for patients with LAPC. However, progression of the primary tumor contributes to more than 30% of deaths and the rate of downstaging is only 4–15%, which suggests the need for novel local control approaches (2, 3).

Irreversible electroporation (IRE) is a novel ablative and non-thermal method (4). IRE was shown to be a suitable treatment for LAPC that encases surrounding vessels because its unique features are not sensitive to the heat sink effect (5, 6). Although the role of concurrent IRE and conventional treatment has been discussed in previous studies, these studies were mostly based on small cohorts of patients and had contradictory results (7, 8). Moreover, prospective data for combination therapy of IRE and chemotherapy in clinical trials is still lacking. It is necessary to fill this gap with new information on the clinical and treatment characteristics of a large cohort of patients with LAPC. In this study, the Surveillance, Epidemiology, and End Results (SEER) database and a study cohort from Sun Yat-sen University Cancer Center (SYSUCC) were adopted to evaluate the efficacy of combination therapy with IRE and chemotherapy in patients with LAPC.

## Materials and Methods

### Patients

The first cohort of patients with LAPC was from the SEER database (2012–2015) of the United States (US) National Cancer Institute, which provided data on cancer incidence and survival in the US and covered 30% of the population. Patients with the following information were included in this study: International Classification of Diseases for Oncology, Third Edition (ICD-O-3) site codes C25.1, C25.2, C25.3, and C25.8. All included patients had pathologically confirmed pancreatic adenocarcinoma or infiltrating ductal carcinoma (ICD-O-3 histology codes 8140/3 and 8500/3, respectively). Only patients who were without distant metastases and whose tumor were classified as T4 were included. The SYSUCC cohort consisted of patients who were initially treated with chemotherapy alone or induction chemotherapy followed by IRE from August 2015 to August 2017. The inclusion criteria were as follows: (1) pathologically confirmed pancreatic adenocarcinoma and radiologically confirmed LAPC. LAPC was defined per the seventh edition of the AJCC staging system for pancreatic cancer, which describes LAPC as arterial encasement of either the celiac axis or superior mesenteric artery or unreconstructable superior mesenteric or portal vein involvement, with no evidence of metastatic disease from abdominal and thoracic computed tomography (CT) (9, 10); (2) four months of induction chemotherapy [FOLFIRINOX or gemcitabine (GEM)-based chemotherapy] without radiotherapy. The following exclusion criteria were adopted: (1) second primary cancer; (2) distant metastases; (3) other treatments, including surgical resection or radiofrequency ablation (RFA); (4) an Eastern Cooperative Oncology Group performance status (ECOG PS) score larger than 2; and (5) missing or incomplete information.

### Data Collection

The SEER database and the medical management system of SYSUCC were used to extract the demographic, clinical, and pathological variables of patients, including age at diagnosis, gender, tumor site, grade and size, tumor-node-metastasis (TNM) stage, ECGO PS score, chemotherapy, radiotherapy, IRE treatment, and cause of death. In addition, other clinical factors, including white blood cell (WBC) count, hemoglobin (HGB), platelet (PLT) count, serum levels of alanine transaminase (ALT), aspartate aminotransferase (AST), alkaline phosphatase (ALP), glutamyltranspeptidase (GGT), albumin (ALB), total bilirubin (TBIL), indirect bilirubin (IBIL), C-reactive protein (CRP), carcinoembryonic antigen (CEA), carbohydrate antigen 19-9 (CA19-9), which were the same as those in our previous reports (11), were also investigated in this study. The endpoints were OS, cancer-specific survival (CSS), and progression-free survival (PFS), which were defined as the duration from the date of treatment to death from all causes, cancer-related deaths, and tumor progression, respectively, or last follow-up. September 30, 2018 was the last date of follow-up.

### Treatment Procedure

Induction chemotherapy of either FOLFIRINOX or GEM-based chemotherapy was used for a duration of 4 months (totaling 3 cycles of GEM-based chemotherapy or 4–6 cycles of FOLFIRINOX-based chemotherapy), which was in accordance with previous studies (12, 13). The same line of chemotherapy was performed 7–14 days after IRE treatment. Patients who did not receive IRE treatment received FOLFIRINOX or GEM-based chemotherapy as the standard treatment. The same procedure of IRE which was reported in our previous research was adopted in the present study (11). During the procedure of IRE, two to six probes will be used according to the size and location of the tumor to create an electric field around the tumor, which will finally cause nanoscale pore formation in the plasma membrane. The generator unit software is used to analysis the probe configuration data of ultrasound and provides optimal voltage and pulse length delivery. A setting of 1,500 V/cm is often used as initial setting, with a planned delivery of 90 pulses at a pulse length of 70–90 ms. Simply, this procedure was similar with the proposed procedure suggested by Martin et al., which was also adopted in cases of SEER database (14, 15).

### Follow-Up

Regular follow-up data was available for each patient: 1 month after IRE for the initial follow-up, and every 2–3 months thereafter. Abdominal CT or MRI, physical examination, and serum CA19-9 and CEA analyses were performed for each follow-up.

### Statistical Analysis

Continuous and categorical variables were compared by student's *t*-test and chi-square test, respectively. The Kaplan-Meier method was used to analyze OS and PFS and the log-rank test was used to compare the survival differences. The cumulative incidence function (CIF) of the variables on cancer- and non-cancer-specific mortality was evaluated by Fine and Gray's model (16, 17). Multivariate analysis was performed using the Cox regression model for variables that were significantly associated with OS, CSS, and PFS in the univariate analysis, to determine the prognostic factors of survival with 95% confidence intervals (CIs) and a significance level of 0.05. Balanced variables were selected by a logistic regression model with propensity score matching (PSM) analysis. One-to-fifteen and one-to-two nearest-neighbor matching algorithms were used in the SEER and SYSUCC databases, respectively (18). PSM results were also reported as effect size: |value| < 0.2 indicated a negligible difference, |value| < 0.5 indicated a small difference, |value| < 0.8 indicated a moderate difference, and any other value indicated a large difference (19, 20). R statistical software (R software version 3.4.2; R Foundation for Statistical Computing, Vienna, Austria) was used to perform all statistical analyses.

## Results

### Patient Characteristics

**Figure 1:**
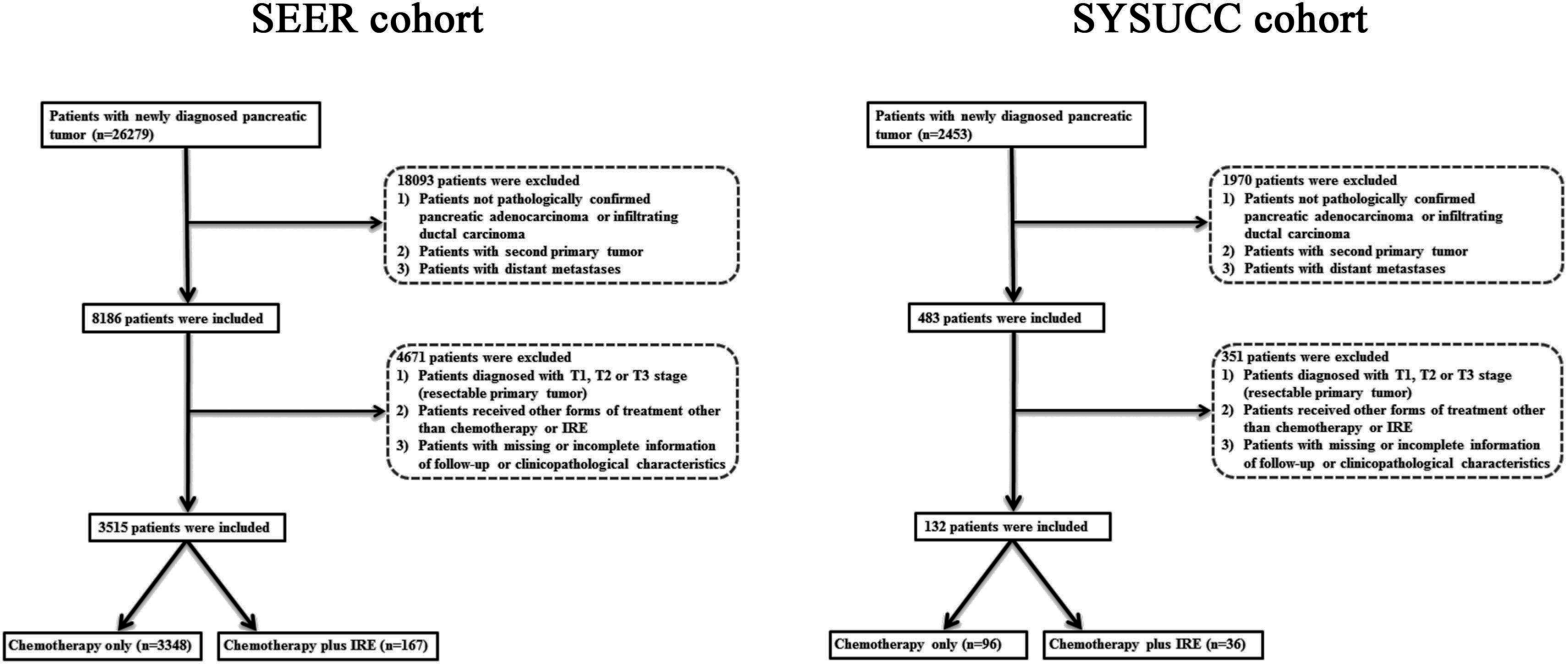
Flow diagram of the data selection process.

The flow diagram for data selection is shown in Figure 1. In total, 3,515 patients with LAPC from SEER database, including 3,348 patients who received chemotherapy only and 167 patients who received combination therapy with IRE and chemotherapy, were included in this study. The baseline clinical and pathological characteristics were compared between the two groups (Table 1). The median age was 65 years (range, 26–97 years) and 67 years (range, 29–96 years) for patients in the combination therapy group and chemotherapy group, respectively. More than half of the tumors were located in the head of the pancreas in both groups. Compared with patients in the combination therapy group, significantly more patients in the chemotherapy group underwent radiotherapy. To equilibrate significantly different baseline characteristics, 167 patients in the combination therapy group and 2,505 matched patients in the chemotherapy group were selected. All variables were balanced after the PSM analysis.

**Table 1:** Comparisons of clinical and imaging characteristics of patients.

**Characteristic**		**Before PSM**				**After PSM**				**Effect size**
		**Chemotherapy +** **IRE**	**Chemotherapy**	**Total number**	***P* value**	**Chemotherapy +** **IRE**	**Chemotherapy**	**Total number**	***P* value**	
**SEER DATASET**										
Total number		167	3,348	3,515		167	2,505	2,672		
Age (years)	≤60	51	946	997	0.538	51	681	732	0.370	0.002
	>60	116	2,402	2,518		116	1,824	1,940		
Gender	Female	78	1,665	1,743	0.476	78	1,251	1,329	0.425	0.001
	Male	89	1,683	1,772		89	1,254	1,343		
Race	Black	16	458	474	0.088	16	351	387	0.084	0.005
	White	141	2,583	2,724		141	1,929	2,070		
	Others	10	307	317		10	225	235		
Tumor size (cm)	≤2	7	120	127	0.778	7	91	98	0.808	0.021
	2~4	71	1,508	1,579		71	1,124	1,195		
	>4	89	1,720	1,809		89	1,290	1,379		
Tumor grade	Well	15	174	189	0.058	15	140	155	0.051	0.016
	Moderate	74	1,522	1,596		74	1,094	1,168		
	Poor	78	1,652	1,730		78	1,271	1,349		
LN metastasis	Absent	119	2,189	2,308	0.133	119	1,654	1,773	0.177	0.007
	Present	48	1,159	1,207		48	851	899		
Tumor site	Head	84	1,799	1,883	0.473	84	1,352	1,436	0.547	0.024
	Body	53	1,057	1,110		53	772	825		
	Tail	30	492	522		30	381	411		
Radiotherapy	No	127	2,119	2,246	0.001	127	1,870	1,897	0.783	0.156
	Yes	40	1,229	1,269		40	635	675		
**SYSUCC DATASET**										
Total number		36	96	132		36	36	72		
Age (years)	≤60	20	52	72	0.887	20	20	40	1.000	0.032
	>60	16	44	60		16	16	32		
Gender	Female	17	71	88	0.006	17	24	41	0.153	0.146
	Male	19	25	44		19	12	31		
Tumor size (cm)	≤2	1	9	10	0.061	1	1	2	0.256	0.032
	2~4	20	34	54		20	13	33		
	>4	15	53	68		15	22	37		
Tumor grade	Well	3	1	4	0.092	3	1	4	0.588	0.047
	Moderate	20	56	76		20	21	41		
	Poor	13	39	52		13	14	27		
LN metastasis	Absent	29	31	60	<0.001	29	29	58	1.000	0.066
	Present	7	65	72		7	7	14		
Tumor site	Head	18	45	63	0.001	18	19	37	0.015	0.014
	Body	15	16	31		15	6	21		
	Tail	3	35	38		3	11	14		
WBC (^*^10^9^)	≤10	32	80	112	0.588	32	34	66	0.674	0.037
	>10	4	16	20		4	2	6		
HGB (g/L)	≤120	10	25	35	0.828	10	10	20	1.000	0.011
	>120	26	71	97		26	26	52		
PLT (^*^10^9^)	≤300	31	77	108	0.613	31	30	61	0.743	0.087
	>300	5	19	24		5	6	11		
ALT (U/L)	≤40	26	70	96	0.936	26	27	53	0.789	0.092
	>40	10	26	36		10	9	19		
AST (U/L)	≤40	29	78	107	0.928	29	31	60	0.753	0.073
	>40	7	18	25		7	5	12		
ALP (U/L)	≤100	18	44	62	0.439	19	18	37	0.816	0.071
	>100	18	52	70		17	18	35		
GGT (U/L)	≤45	19	37	56	0.166	19	19	38	0.989	−0.001
	>45	17	59	76		17	17	34		
ALB (g/L)	≤40	4	42	46	<0.001	4	10	14	0.135	0.110
	>40	32	54	86		32	26	58		
TBIL (umol/L)	≤20.5	27	76	103	0.640	27	27	54	1.000	0.013
	>20.5	9	20	29		9	9	18		
IBIL (umol/L)	≤15	32	93	125	0.099	32	36	68	0.115	−0.182
	>15	4	3	7		4	0	4		
CRP (ng/L)	≤3	24	30	54	<0.001	25	20	45	0.216	0.072
	>3	12	66	78		11	16	27		
CEA (ng/mL)	≤5	21	40	61	0.116	21	15	36	0.157	0.175
	>5	15	56	71		15	21	36		
CA19-9 (U/ml)	≤35	9	13	22	0.189	9	5	14	0.372	−0.212
	>35	27	83	110		27	30	57		
HBsAg	Negative	33	88	121	0.974	33	33	66	1.000	0.003
	Positive	3	8	11		3	3	6		
ECGO PS score	0	15	41	56	0.809	15	20	35	0.468	0.165
	1	19	52	71		19	15	34		
	2	2	3	5		2	1	3		
Chemotherapy	FOLFIRINOX	21	52	73	0.668	22	23	45	0.796	0.087
	Gem	15	44	59		14	13	27		

*LN, lymph node metastasis; TNM, tumor-node-metastasis stage; WBC, white blood cell count; PLT, platelet count; ALT, alanine transaminase; AST, aspartate aminotransferase; ALP, alkaline phosphatase; GGT, glutamyl transpeptidase; ALB, albumin; TBIL, total bilirubin; IBIL, indirect bilirubin; CRP, C-reactive protein; HBsAg, hepatitis B surface antigen; CEA, carcinoembryonic antigen; CA19-9, carbohydrate antigen 19-9; HBsAg, hepatitis B surface antigen; ECOG PS, Eastern Cooperative Oncology Group (ECOG) performance status*.

Additionally, 132 patients from the SYSUCC were included. The median age for patients in the combination therapy group and the chemotherapy group was 60 years (range, 39–80 years) and 60 years (range, 45–87 years), respectively. Female patients, patients with tumors larger than 4 cm or those with moderately differentiated tumors were commonly seen in both groups. The two groups of patients had substantially balanced variables after the PSM analysis.

### OS Comparison Between the Two Groups

**Figure 2:**
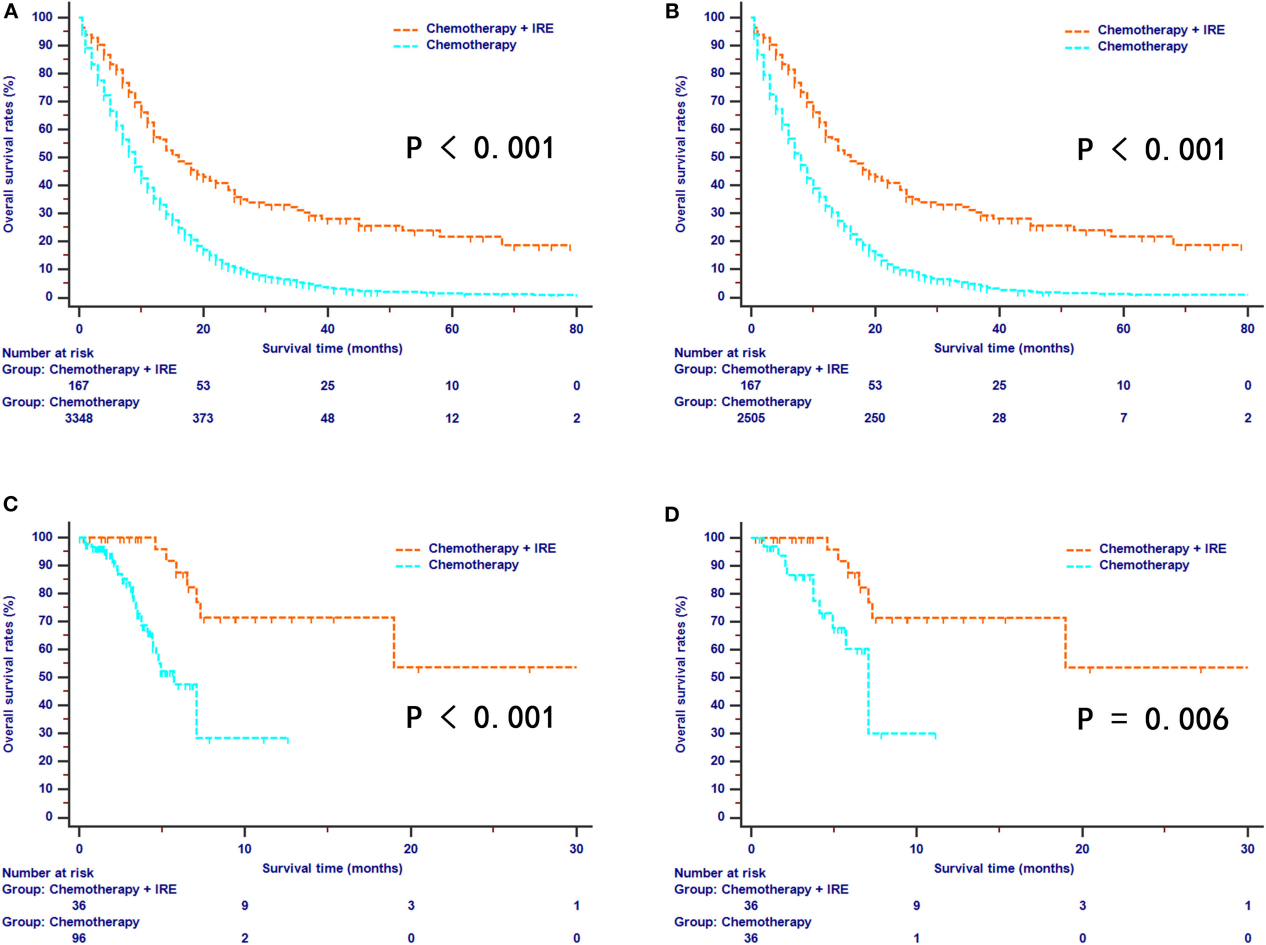
The survival curves of overall survival stratified by treatment strategies for patients with LAPC from SEER dataset before **(A)** and after **(B)** propensity score matching. The survival curves of overall survival stratified by treatment strategies for patients with LAPC from SYSUCC dataset before **(C)** and after **(D)** propensity score matching.

For the SEER series, survival curves for OS were well-separated between the combination therapy and chemotherapy groups. The median OS was 16 months (95% CI, 12–21 months) for the combination therapy group and 9 months (95% CI, not available) for the chemotherapy group (Figure 2A). After the PSM analysis, combination therapy provided a more obvious survival benefit than chemotherapy alone (median OS, 16 months vs. 8 months; 1-year OS rates, 57.1% vs. 32.4%; 3-year OS rates, 30.2% vs. 4.4%; 5-year OS rates, 21.8% vs. 0.9%, *P* < 0.001, Figure 2B). For the SYSUCC series, survival curves were also well-separated by the different treatments. During the follow-up period, the median OS for the combination therapy group was 21.6 months while the median OS for the chemotherapy group was 7.1 months (Figure 2C). In addition, compared with the chemotherapy group, the combination therapy group experienced significantly higher survival rates (1-year OS rates, 71.4% vs. 30.1%; 2-year OS rates, 53.5% vs. 30.1%, *P* = 0.006, Figure 2D).

### CSS and PFS Comparisons Between the Two Groups

**Figure 3:**
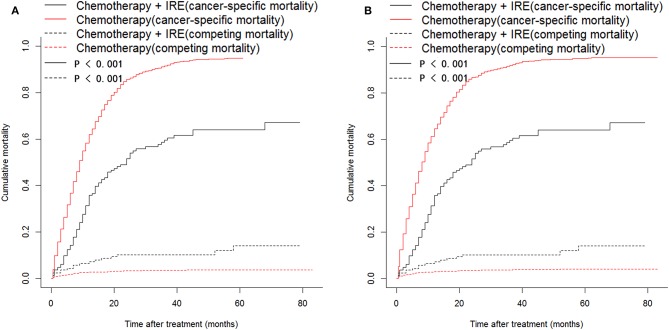
Cumulative cancer-specific and competing mortalities stratified by treatment strategies for patients with LAPC from SEER dataset before **(A)** and after **(B)** propensity score matching.

**Figure 4:**
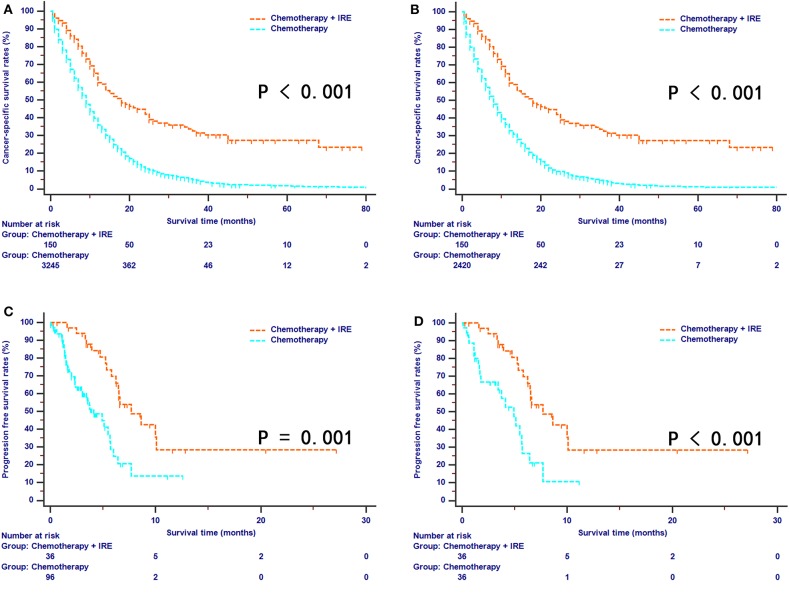
The survival curves of cancer-specific survival stratified by treatment strategies for patients with LAPC from SEER dataset before **(A)** and after **(B)** propensity score matching. The survival curves of progression-free survival stratified by treatment strategies for patients with LAPC from SYSUCC dataset before **(C)** and after **(D)** propensity score matching.

For the SEER series, a total of 2,844 out of 3,348 (84.9%) patients died during the follow-up period, which was more than 80 months. Among these deaths, there were 87 (52.1%) cancer-specific and 17 (10.2%) non-cancer-specific deaths for the combination therapy group and 2,637 (78.8%) cancer-specific and 103 (3.1%) non-cancer-specific deaths for the chemotherapy group. The comparison of cancer-specific mortality and non-cancer-specific mortality is shown in Figure 3. Patients in the chemotherapy group were at higher risk of the cumulative incidence of higher cancer-specific mortality than those in the combination therapy group (Figure 4A). Compared with the chemotherapy group, the combination therapy group experienced significantly higher CSS rates (median CSS, 18 months vs. 8 months; 1-year CSS rates, 59.7% vs. 32.9%; 3-year CSS rates, 32.5% vs. 4.4%; 5-year CSS rates, 27.3% vs. 1.0%, *P* < 0.001, Figure 4B). For the SYSUCC series, 15 (41.7%) patients experienced disease progression in the combination therapy group, while 42 (43.8%) patients experienced disease progression in the chemotherapy group. After PSM, a higher proportion of patients (19, 52.8%) experienced disease progression in the chemotherapy group compared with that (15, 41.7%) in the combination therapy group. The median PFS for patients was 7.7 months (95% CI, 6.2–10.1 months) in the combination therapy group and 4.1 months (95% CI, 3.1–5.7 months) in the chemotherapy group (*P* < 0.001, Figure 4C). Similar results were also obtained for patients after PSM (*P* = 0.001, Figure 4D). Patients in the chemotherapy group were 2.67 times more likely to experience tumor progression compared with those in the combination therapy group.

### Prognostic Factors for OS, CSS, and PFS

Univariate and multivariate analyses were conducted for OS, CSS, and PFS in this study. For the SEER database, a univariate analysis illustrated that age, tumor size, tumor grade, radiotherapy, and chemotherapy combined with IRE were associated with OS and CSS for patients with LAPC. Furthermore, a multivariate model illustrated that chemotherapy combined with IRE was associated with increased OS and CSS. Other significant independent unfavorable prognostic factors for OS included age older than 60 years [hazard ration (HR) 1.283, 95% CI 1.166–1.412, *P* < 0.001], poor tumor differentiation (HR 1.081, 95% CI 1.007–1.160, *P* = 0.032), tumor size larger than 4 cm (HR 1.138, 95% CI 1.056–1.226, *P* = 0.001), and history of radiotherapy (HR 0.608, 95% CI 0.552–0.671, *P* < 0.001) (Table 2). The same factors were identified as significant prognostic factors for CSS in patients with LAPC (Table 3).

**Table 2:** Univariate and multivariate analyses of OS in patients.

**Characteristic**		**Before PSM**						**After PSM**					
		**Univariate analysis**			**Multivariate analysis**			**Univariate analysis**			**Multivariate analysis**		
		**HR**	**95% CI**	***P***	**HR**	**95% CI**	***P***	**HR**	**95% CI**	***P***	**HR**	**95% CI**	***P***
**SEER DATASET**													
Age (years)	≤60/>60	1.295	1.193–1.406	<0.001	1.281	1.180–1.391	<0.001	1.304	1.186–1.435	<0.001	1.283	1.166–1.412	<0.001
Gender	Female/Male	0.999	0.928–1.075	0.984			NI	0.994	0.914–1.082	0.895			NI
Race	Black/White/Others	0.949	0.876–1.027	0.194			NI	0.937	0.855–1.026	0.159			
Tumor size (cm)	≤2/2~4/>4	1.137	1.066–1.213	<0.001	1.148	1.075–1.225	<0.001	1.135	1.054–1.222	0.001	1.138	1.056–1.226	0.001
Tumor grade	Well/Moderate/Poor	1.115	1.048–1.186	0.001	1.077	1.012–1.147	0.019	1.119	1.043–1.200	0.002	1.081	1.007–1.160	0.032
LN metastasis	Absent/Present	1.076	0.996–1.162	0.064			NI	1.072	0.981–1.172	0.123			NI
Tumor site	Head/Body/Tail	0.956	0.909–1.006	0.082			NI	0.960	0.960–1.016	0.157			NI
Radiotherapy	No/Yes	0.640	0.592–0.691	<0.001	0.610	0.565–0.660	<0.001	0.630	0.572–0.694	<0.001	0.608	0.552–0.671	<0.001
Chemotherapy	Without IRE/With IRE	0.428	0.351–0.522	<0.001	0.369	0.302–0.451	<0.001	0.403	0.329–0.492	<0.001	0.370	0.302–0.453	<0.001
**SYSUCC DATASET**													
Age (years)	≤60/>60	1.154	0.600–2.222	0.668			NI	0.889	0.351–0.253	0.804			NI
Gender	Female/Male	2.399	1.077–5.343	0.052			NI	4.630	1.317–16.275	0.017	4.975	1.081–22.891	0.039
Tumor size (cm)	≤2/2~4/>4	1.657	0.843–3.257	0.143			NI	2.863	1.021–8.033	0.046	2.012	0.764–5.294	0.157
Tumor grade	Well/Moderate/Poor	1.182	0.669–2.086	0.565			NI	1.797	0.680–3.293	0.316			NI
LN metastasis	Absent/Present	7.966	3.285–19.315	<0.001	4.091	1.484–11.278	0.006	7.264	2.220–23.775	0.001	4.799	1.173–19.625	0.029
Tumor site	Head/Body/Tail	1.317	0.879–1.973	0.182			NI	1.310	0.700–2.452	0.398			NI
WBC (^*^10^9^)	≤10/>10	1.058	0.371–3.019	0.916			NI	0.463	0.061–3.527	0.457			NI
HGB (g/L)	≤120/>120	0.852	0.419–1.733	0.659			NI	1.401	0.461–4.264	0.552			NI
PLT (^*^10^9^)	≤300/>300	0.513	0.181–1.455	0.209			NI	0.484	0.110–2.126	0.337			NI
ALT (U/L)	≤40/>40	0.929	0.435–1.981	0.848			NI	1.034	0.365–2.929	0.950			NI
AST (U/L)	≤40/>40	1.006	0.417–2.428	0.989			NI	0.623	0.143–2.719	0.529			NI
ALP (U/L)	≤100/>100	1.686	0.867–3.277	0.124			NI	1.395	0.549–3.546	0.484			NI
GGT (U/L)	≤45/>45	1.646	0.840–3.224	0.146			NI	2.106	0.821–5.400	0.121			NI
ALB (g/L)	≤40/>40	0.261	0.133–0.515	0.101			NI	0.437	0.153–1.244	0.121			NI
TBIL (umol/L)	≤20.5/>20.5	0.712	0.296–1.715	0.449			NI	0.360	0.083–1.569	0.174			NI
IBIL (umol/L)	≤15/>15	0.354	0.048–2.589	0.306			NI	0.043	0.001–77.525	0.411			NI
CRP (ng/L)	≤3/>3	3.312	1.582–6.936	0.001	1.741	0.757–4.005	0.192	3.094	1.136–8.428	0.127			NI
CEA (ng/mL)	≤5/>5	1.029	0.527–2.011	0.933			NI	1.264	0.495–3.232	0.624			NI
CA19-9 (U/ml)	≤35/>35	1.745	0.676–4.507	0.250			NI	1.714	0.494–5.951	0.396			NI
HBsAg	Negative/positive	0.220	0.030–1.610	0.136			NI	0.264	0.094–0.738	0.011			NI
Chemotherapy	Without IRE/with IRE	0.206	0.082–0.515	0.001	0.363	0.132–0.998	0.050	0.264	0.094–0.738	0.011	0.313	0.098–0.992	0.048
Chemotherapy type	FOLFIRINOX/Gem	0.910	0.648–1.277	0.584			NI	0.852	0.513–1.414	0.535			NI

*OS, overall survival; HR, hazard ratio; NI, not included; Other abbreviations as in Table 1*.

**Table 3:** Univariate and multivariate analyses of CSS and PFS in patients.

**Characteristic**		**Before PSM**						**After PSM**					
		**Univariate analysis**			**Multivariate analysis**			**Univariate analysis**			**Multivariate analysis**		
		**HR**	**95% CI**	***P***	**HR**	**95% CI**	***P***	**HR**	**95% CI**	***P***	**HR**	**95% CI**	***P***
**CSS** **SEER DATASET**													
Age (years)	≤60/>60	1.282	1.179–1.394	<0.001	1.269	1.167–1.381	<0.001	1.278	1.160–1.408	<0.001	1.258	1.142–1.387	<0.001
Gender	Female/Male	0.998	0.926–1.076	0.957			NI	0.988	0.906–1.077	0.784			NI
Race		0.963	0.888–1.045	0.368			NI	0.955	0.870–1.048	0.328			NI
Tumor size (cm)	≤2/2~4/>4	1.128	1.056–1.204	<0.001	1.161	1.086–1.243	<0.001	1.125	1.043–1.213	0.002	1.129	1.046–1.219	0.002
Tumor grade	Well/Moderate/Poor	1.114	1.046–1.187	0.001	1.167	1.082–1.260	<0.001	1.116	1.039–1.200	0.003	1.079	1.004–1.161	0.040
LN metastasis	Absent/Present	1.090	1.007–1.179	0.032	1.069	0.988–1.157	0.099	1.088	0.994–1.192	0.069			NI
Tumor site	Head/Body/Tail	0.950	0.902–1.000	0.050	0.884	0.830–0.941	<0.001	0.951	0.897–1.009	0.095			NI
Radiotherapy	No/Yes	0.647	0.598–0.700	<0.001	0.614	0.567–0.664	NI	0.635	0.575–0.701	<0.001	0.611	0.553–0.675	<0.001
Chemotherapy	Without IRE/With IRE	0.372	0.300–0.462	<0.001	0.332	0.267–0.413	<0.001	0.351	0.283–0.437	<0.001	0.323	0.260–0.403	<0.001
**PFS** **SYSUCC DATASET**													
Age (years)	≤60/>60	0.690	0.410–1.163	0.164			NI	0.728	0.379–1.397	0.340			NI
Gender	Female/Male	1.513	0.868–2.639	0.144			NI	1.692	0.864–3.315	0.125			NI
Tumor size (cm)	≤2/2~4/>4	1.375	0.848–2.232	0.196			NI	1.537	0.827–2.856	0.174			NI
Tumor grade	Well/Moderate/Poor	1.271	0.814–1.986	0.291			NI	1.226	0.724–2.077	0.447			NI
LN metastasis	Absent/Present	2.867	1.588–5.176	<0.001	2.380	1.269–4.460	0.007	3.190	1.526–6.672	0.002	4.170	1.898–9.163	<0.001
Tumor site	Head/Body/Tail	1.281	0.930–1.763	0.130			NI	1.388	0.900–2.142	0.138			NI
WBC (^*^10^9^)	≤10/>10	1.282	0.628–2.616	0.495			NI	0.995	0.315–2.823	0.993			NI
HGB (g/L)	≤120/>120	1.155	0.642–2.077	0.631			NI	1.311	0.620–2.774	0.479			NI
PLT (^*^10^9^)	≤300/>300	0.819	0.412–1.628	0.570			NI	0.512	0.198–1.323	0.167			NI
ALT (U/L)	≤40/>40	0.753	0.413–1.374	0.355			NI	0.733	0.346–1.550	0.416			NI
AST (U/L)	≤40/>40	0.775	0.381–1.579	0.483			NI	0.841	0.350–2.018	0.697			NI
ALP (U/L)	≤100/>100	1.024	0.614–1.709	0.927			NI	0.956	0.506–1.808	0.890			NI
GGT (U/L)	≤45/>45	0.870	0.518–1.459	0.597			NI	0.729	0.376–1.411	0.348			NI
ALB (g/L)	≤40/>40	0.775	0.445–1.349	0.367			NI	0.750	0.354–1.587	0.452			NI
TBIL (umol/L)	≤20.5/>20.5	0.544	0.266–1.112	0.095			NI	0.419	0.174–1.008	0.052			NI
IBIL (umol/L)	≤15/>15	1.116	0.445–2.804	0.815			NI	0.796	0.244–2.600	0.705			NI
CRP (ng/L)	≤3/>3	1.605	0.922–2.795	0.095			NI	1.419	0.696–2.893	0.336			NI
CEA (ng/mL)	≤5/>5	1.123	0.668–1.890	0.662			NI	1.221	0.643–2.320	0.542			NI
CA19-9 (U/ml)	≤35/>35	1.965	0.927–4.167	0.078			NI	3.258	1.148–9.247	0.027	1.191	0.394–3.606	0.757
HBsAg	Negative/Positive	0.501	0.181–1.393	0.185			NI	0.805	0.246–2.634	0.719			NI
Chemotherapy	Without IRE/With IRE	0.357	0.197–0.649	0.001	0.491	0.251–0.960	0.038	0.350	0.180–0.678	0.002	0.305	0.146–0.638	0.002
Chemotherapy type	FOLFIRINOX/Gem	1.006	0.781–1.298	0.960			NI	0.996	0.719–1.380	0.980			NI

*CSS, cancer-specific survival; PFS, progression-free survival; HR, hazard ratio; Other abbreviations as in Table 1*.

The data from the SYSUCC series were also used to analyze the prognostic factors of OS and PFS. Gender, tumor size, lymph node (LN) metastasis and chemotherapy plus IRE were associated with OS in patients with LAPC. In addition, after a stepwise removal of variables in the multivariate analysis, male gender (HR 4.975, 95% CI 1.081–22.891, *P* = 0.039), presence of LN metastasis (HR 4.799, 95% CI 1.173–19.625, *P* = 0.029), and chemotherapy plus IRE (HR 0.313, 95% CI 0.098–0.992, *P* = 0.048) were identified as independent prognostic factors for OS (Table 2). A multivariate analysis revealed that chemotherapy plus IRE was identified as an independent favorable prognostic factor for PFS in patients in both the entire cohort and the matched cohort (Table 3).

## Discussion

Previous studies have shown a survival benefit for IRE plus conventional treatment in patients with LAPC (6, 7, 21). Most of the aforementioned studies were single-arm studies. There was one study that suggested that treatment with IRE combined with chemotherapy increased the median OS of patients with LAPC compared to conventional treatments (22). However, all these studies contained limited patient cohorts or lacked external validation. In contrast, another small cohort and single-center study showed no significant survival benefit from IRE after induction chemotherapy (8). The significance of the lack of rigorous quality assessments in these studies in regard to their negative outcomes is unknown. Multiple smaller studies with varying chemotherapy regimens have provided mixed results (6, 8, 23). Therefore, there has been no strong evidence for the survival benefit of IRE combined with chemotherapy in LAPC patients. The current study compared the efficacy between IRE plus chemotherapy and chemotherapy in patients with LAPC based on the cohorts from the SEER database and the SYSUCC database, a “real-world” dataset. To our knowledge, this is the largest study evaluating the role of IRE combined with chemotherapy in patients with LAPC. Our analysis provided evidence that patients with LAPC who were treated with IRE combined with chemotherapy had a more obvious survival benefit compared with those who were treated with chemotherapy alone.

In this study, all patients in the SYSUCC dataset received standard chemotherapy, including GEM-based chemotherapy or FOLFIRINOX-based chemotherapy. In addition, gemcitabine-based chemotherapy has been the first-line therapy for advanced pancreatic cancer since the positive survival results of gemcitabine-based chemotherapy were revealed in 1997 (24). It can be assumed that the chemotherapy regimen, which was mainly GEM-based chemotherapy for patients from the SEER database, was uniform and recommended for patients with LAPC during this time period (24, 25). The median OS was 16 and 21.6 months for patients after IRE combined with chemotherapy in the SEER and SYSUCC datasets, respectively. The median OS was similar to that from a study conducted by Huang et al. (26) and was significantly higher than that of patients after chemotherapy alone (8.0 months for the SEER patients and 7.1 months for the SYSUCC patients). In addition, the SEER patients had a long follow-up period, and it was shown that combination therapy was superior to chemotherapy alone with respect to not only 5-year OS rates but also 5-year CSS rates for all enrolled patients even though larger proportions of patients in the chemotherapy group received radiotherapy. After the PSM analysis, the advantages of survival benefits in terms of OS and CSS from combination therapy were even more obvious, compared with those from chemotherapy alone in both the SEER and SYSUCC patients. In the present study, 21 patients received FOLFIRINOX-based chemotherapy in the SYSUCC cohort, although patients with LAPC had significantly improved survival from the newest modified FOLFIRINOX-based chemotherapy (27). FOLFIRINOX-based chemotherapy became popular more recently. Only a proportion of patients received FOLFIRINOX-based chemotherapy in this study. The absence of uniform protocols and the intolerance to the full dosages of FOLFIRINOX-based chemotherapy might partially explain the unsatisfactory results for chemotherapy in this study. In addition, cross-study comparisons were also conducted. Studies conducted by Krishnan et al. (28) and Huguet et al. (29) showed that chemotherapy combined with radiotherapy contributed to the median OS, which was nearly 12 months for patients with LAPC, and significantly lower than that of patients who received IRE combined with chemotherapy. These comparisons further consolidated the survival advantages of combination therapy over chemotherapy alone. In addition, there was a disparity in the survival between the SEER and SYSUCC patients, the development of chemotherapy regimens and technology of IRE may ultimately improve the survival of LAPC patients after combination therapy. However, a survival benefit of IRE combined with chemotherapy was observed in patients with LAPC who received any type of chemotherapy. Radiotherapy was also shown as a favorable prognostic factor for both OS and CSS for patients with LAPC after IRE plus chemotherapy therapy. The enhanced local and systemic control of diseases further improved survival outcomes, showing that maybe IRE was an effective supplement to the standard therapy for LAPC patients.

Tumor progression was commonly observed in patients with LAPC even after treatment. Compared with the 19 out of 36 (52.8%) patients who experienced disease progression after chemotherapy, 15 out of 36 (41.7%) patients experienced disease progression after IRE combined with chemotherapy during the follow-up period. Compared with chemotherapy alone, IRE combined with chemotherapy resulted in significantly longer PFS. IRE assists the chemotherapy delivery to the tumor by disrupting the dense stroma of pancreatic cancer (30, 31). Another mechanism of IRE is the formation of nanoscale micropores in the lipid bilayer of cell membranes increases its permeability to macromolecules, including chemical (12). Moreover, in IRE treatment, an electric field surrounds the entire tumor without causing thermal damage to important nearby structures (12). Maybe these mechanisms help to explain the synergism of IRE and chemotherapy compared with chemotherapy alone. In addition, as a local destructive treatment, maybe IRE could only control the local disease, the subsequent chemotherapy was needed to adequately control microscopic disease. IRE combined with chemotherapy created a synergistic effect to decrease the local recurrence rates and to lengthen PFS for patients with LAPC.

Similar to another study with a large cohort conducted by Martin et al. (32), which was a single-arm study showing an encouraging 24.9-month median OS and 12.4-month median PFS, our study provided new data illustrating the survival benefit from IRE combined with chemotherapy. In contrast to our study, 25% of patients in Martin's study underwent resection and IRE margin accentuation. Our study focused on patients which were not suitable for concomitant resection and aimed to provide new insights on this combination therapy for patients with LAPC. In addition, with the large cohort from the SEER database and the external cohort from the SYSUCC database, a survival benefit for IRE combined with chemotherapy was clearly shown, which suggests that IRE combined with chemotherapy was an effective multimodal approach and assisted both local tumor reduction and systemic control of the disease in patients with LAPC.

This study has limitations. First, there was potential selection bias in this retrospective trial. The PSM analysis decreased the selection bias associated with the variables incorporated into the comparisons, but these biases could not be completely removed. The SEER database provided a sufficient sample size to eliminate heavily selected observations without reducing power to robustly evaluate treatment effects. However, despite such matching, residual confounding by indication due to unmeasured variables cannot be ruled out. Second, detailed information on chemotherapy, including the dose and course of chemotherapy, and some important hematological indexes, such as CA19-9 and CEA, are not available in the SEER database. Third, there was no information on the comorbidities or performance status of the patients in this study. Although strict inclusion criteria were followed and only patients who received IRE treatment were included, the sample size of patients from the SYSUCC database was small, and the follow-up period was not long enough to make definitive conclusions. Therefore, the survival benefit of IRE in patients with LAPC needs to be validated in another large cohort.

In conclusion, compared with chemotherapy alone, IRE combined with chemotherapy contributed to better long-term survival in patients with LAPC. A randomized clinical trial comparing the efficacy of IRE and chemotherapy is therefore warranted.

## Data Availability Statement

The datasets generated for this study are available on request to the corresponding author.

## Ethics Statement

This study was approved by the Institutional Review Board (IRB) of the Sun Yat-sen University Cancer Center. Each individual participant from SYSUCC database has signed the informed written consent. All procedures performed in studies involving human participants were in accordance with the 1964 Helsinki Declaration and its later amendments or comparable ethical standards.

## Author Contributions

SL designed the project, reviewed, and edited the manuscript, respectively. CH, XH, and YZ performed the study selection, data extraction, statistical analyses, and wrote the main manuscript. CH, XH, YZ, ZC, and XL contributed in classification criteria discussion. All authors reviewed the manuscript.

### Conflict of Interest

The authors declare that the research was conducted in the absence of any commercial or financial relationships that could be construed as a potential conflict of interest.
